# Malignant peritoneal mesothelioma—a diagnostic challenge

**DOI:** 10.1093/jscr/rjac555

**Published:** 2022-12-07

**Authors:** Saad Abdul Razzak, Faisal Awan, Salman Ahmed

**Affiliations:** General Surgery Department, St. Luke’s General Hospital, Kilkenny, Ireland; St Luke’s General Hospital, Kilkenny, Ireland; General Surgery Department, St. Luke’s General Hospital, Kilkenny, Ireland

## Abstract

Malignant peritoneal mesothelioma is a rare cancer originating primarily from the peritoneum with a poor prognosis and non-specific clinical presentation. We present a case of a 60-year-old male, retired metallurgy engineer who initially presented with shortness of breath, lethargy, weight loss, vague abdominal pain and night sweats. Extensive workup for almost 2 months finally leads to the diagnosis of primary malignant peritoneal mesothelioma based on immunohistochemical analysis of loss of BAP1 *gene*. The patient was deemed non-suitable for surgical management and started on palliative carboplatin and pemetrexed. In conclusion, histological diagnosis is essential for peritoneal diseases before considering it as a metastasis from other primary tumours. Furthermore, immunohistochemical analysis and genetic profiling may also guide towards the diagnosis and possible treatment.

## INTRODUCTION

Malignant mesothelioma can be originated from different sites including the pleura, peritoneum, pericardium and tunica vaginalis of the testes. The incidence of mesothelioma in the USA is estimated to be 1 in 100 000 people. 85% of males are attributable to occupational exposure to asbestos. High-risk occupations include mechanics, shipyard workers, plumbers, electricians and roofers. Insidious neoplasm with poor prognosis—long latency period of up to 40 years. Malignant peritoneal mesothelioma accounts for up to 15–20% of all cases of mesothelioma. Patients are younger at diagnosis and have a less clear association with asbestos.

## CASE PRESENTATION

A 60-year-old gentleman, a retired metallurgy engineer, presented himself in early September 2021 with complaints of shortness of breath, weight loss, lethargy, vague abdominal pain, distension and night sweats for the last couple of months. He has no significant past medical or surgical history, a non-smoker with no relevant family history of cancers.

**Figure 1:**
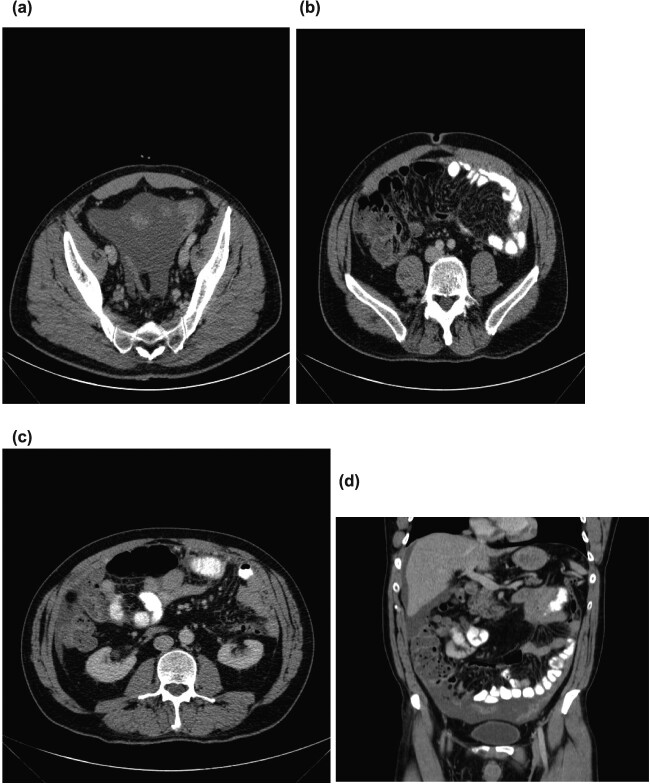
(**a**) Pelvic ascites. (**b**) Axial section showing omental caking. (**c**) Axial section showing omental caking. (**d**) Sagittal section showing peritoneal nodularity and ascites.

On initial examination, he was pale looking, afebrile and vitally stable; the abdomen was soft but distended. His initial blood investigations revealed microcytic hypochromic anaemia with haemoglobin of 9.2 mg/dl, low serum iron, transferrin, and ferritin, and high CRP (187 mg/l). Further workup was started with gastroscopy and colonoscopy, which were normal, followed by CT-TAP that showed multiple mesenteric implants with omental caking and gross ascites. No primary tumour localization was identified (Fig. 1a–d).

**Figure 2:**
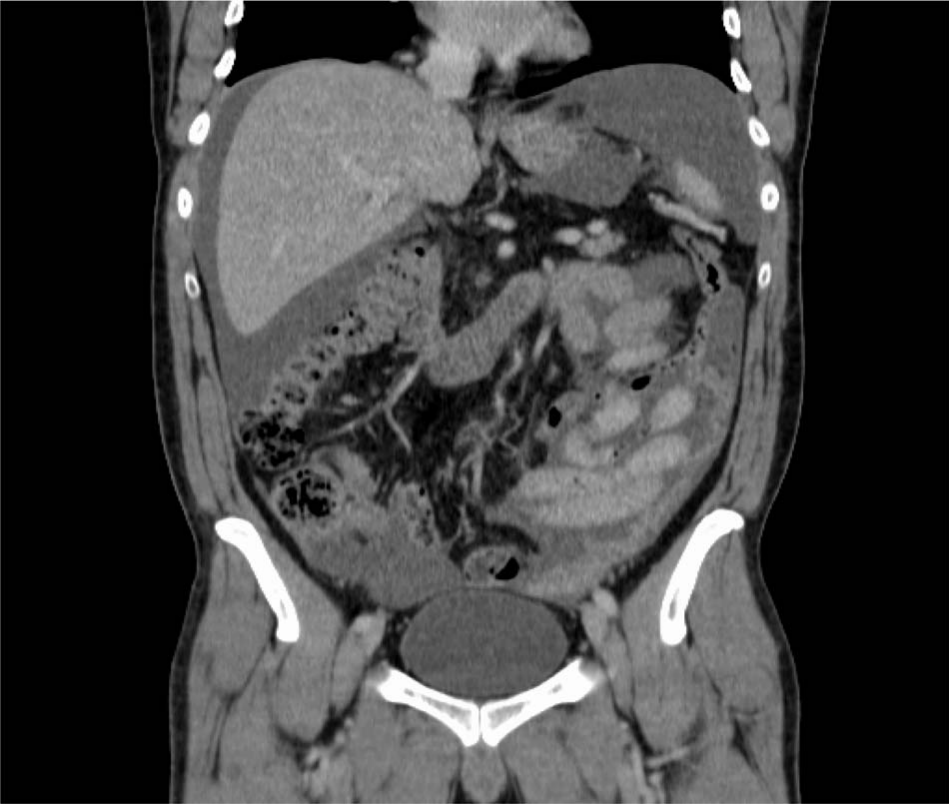
Increase in the amount of ascites post-ultrasound-guided biopsy.

After discussion in radiology MDT, an ultrasound-guided omental implant biopsy was taken. Post-procedure, he developed abdominal pain, fever and increasing CRP. Histology showed cores comprising bowel walls, chronic inflammatory infiltrates and numerous plasma cells and mesothelial cells in serosa, but no malignancy was seen. Repeat CT-AP reported a slight increase in the volume of ascites with mild peritoneal enhancement in the pelvis suggesting early/mild peritonitis; however, no free air was identified (Fig. 2).

Due to clinical deterioration, a diagnostic laparoscopy was performed, which identified haemorrhagic ascites, omental caking in the pelvis, supracolic and pelvic compartments completely frozen. Omental biopsies were taken, and ascites fluid was sent for cytology. Ascitic fluid cytology was negative for malignant cells, and tumour markers were normal. Workup for Tuberculosis was also negative. Omental tissue histology reported florid mesothelial proliferation—uncertain for reactive or neoplastic process. A reactive lymphoid component within the adipose tissue stroma was identified but no histological or immunohistochemical evidence of metastatic carcinoma. For further confirmation due to indistinct findings, samples were sent to a higher speciality centre, which also reported similar findings. Furthermore, BAP 1 staining was suggested, which unfortunately was not available in the country; therefore, samples were sent abroad. The following report shows atypical mesothelial proliferation with occasional mitosis and loss of MTAP staining and nuclear loss of BAP1, which confirms the diagnosis of EPITHELIOD MESOTHELIOMA.

His case was discussed in mesothelioma MDT and the decision for systemic chemotherapy with carboplatin and pemetrexed was made due to his extensive nature of disease burden.

CT-TAP after four cycles of chemotherapy showed a reduction in ascites, new bilateral pleural effusion and sub-centimetre pulmonary nodularity. He has improved significantly, therefore, continued pemetrexed. CT-AP was recently repeated after almost a year of initial diagnosis, which shows some disease progression but he is able to maintain his functionality.

## DISCUSSION

Malignant mesothelioma can be originated from different sites including the pleura, peritoneum, pericardium and tunica vaginalis of the testes. The incidence of mesothelioma in the USA is estimated to be 1 in 100 000 people in the states with no asbestos industry and 2–3 cases per 100 000 persons in states with an asbestos industry. The use of asbestos massively reduced through the 1970s and was banned in the UK in 1999 and in the EU in 2005. High-risk occupations include mechanics, shipyard workers, plumbers, electricians and roofers. It is an insidious neoplasm with a poor prognosis—a long latency period of up to 40 years.

Based on histopathology, mesothelioma can be divided into three subtypes: epithelioid—60% with the best prognosis, sarcomatoid—20% and biphasic—20%. Malignant peritoneal mesothelioma accounts for up to 15–20% of all cases of mesothelioma. Patients are younger at diagnosis with a male to female predominance of 1:1, which may be since there is less association with occupational exposure. Epithelioid represents up to 90% of cases of MPM and has the best prognosis, 25% biphasic and sarcomatoid extremely rare.

Computed tomography (CT) scanning is the most utilized staging modality; however, magnetic resonance imaging with specific acquisition protocols may be increasingly used in future. Radiographic imaging may reveal solid, heterogenous, soft tissue mass with irregular margins and demonstrate high contrast between the enhanced tumour and non-enhanced ascites. Peritoneal and mesenterial thickening may also be noted. The role of positron emission tomography-CT still needs to be defined.

Median survival in MPM ranges from 7 to 13.5 months [1]. Patients who had systemic chemotherapy had shown survival of up to 29 months [2]. In 2015, Carbone’s team identified that patient with mesothelioma having germline mutations has a 7-fold increased survival [3]. The team assessed 79 patients with a family history of mesothelioma and/or other cancers and identified that 43 of 79 patients had deleterious germline BAP1 mutations: their median survival was 5 years. Among the remaining, other mutations were identified like MLH1 (lynch syndrome), and TP53 (Li–Fraumeni syndrome), with a median survival of 9 years [4]. Mesotheliomas carrying BAP1 mutations are almost always of epithelioid variety, well-differentiated and consistent with overall good survival. It is an attractive therapeutic target and prognostic biomarker because it is the most frequently mutated gene in mesothelioma [5].

With the evidence of peritoneal disease burden, metastasis from ovarian cancer in females and from the GI tract in males is usually considered. However, the possibility of primary malignant peritoneal mesothelioma should not be overlooked. Therefore, histological diagnosis with an image-guided core biopsy or preferably a laparoscopic omental or peritoneal deposit biopsy should always be considered essential.

In this case, the importance of immunohistochemistry, especially the detection of BAP1 mutation, can be emphasized in reaching the diagnosis of malignant peritoneal mesothelioma. Also, its non-availability can lead to delay in the diagnosis, which not only hinders the management but also leads to an increase in patient and family’s psychological stress. Furthermore, with a confirmed diagnosis of MPM, an appropriate management plan can be made that may lead to a more conservative approach to extensive disease burden rather than rendering patients with incomplete (R1/R2) resection which confers no additional benefit on overall survival.

## CONCLUSION

This case report identifies the importance of immunohistochemical testing and genetic profiling in the diagnosis of primary peritoneal mesothelioma, especially BAP 1 mutation, and also defines the pathway for the diagnosis and management of mesothelioma.

## References

[1] Kaya H , Sezgi C, Tanrikulu AC, Taylan M, Abakay O, Sen HS, et al. Prognostic factors influencing survival in 35 patients with malignant peritoneal mesothelioma. Neoplasma 2014;61:433–8. 24645844 10.4149/neo_2014_053

[2] Eltabbakh GH , Piver MS, Hempling RE, Recio FO, Intengen ME. Clinical picture, response to therapy, and survival of women with diffuse malignant peritoneal mesothelioma. J Surg Oncol 1999;70:6–12. 9989414 10.1002/(sici)1096-9098(199901)70:1<6::aid-jso2>3.0.co;2-x

[3] Baumann F , Flores E, Napolitano A, Kanodia S, Taioli E, Pass H, et al. Mesothelioma patients with germline BAP1 mutations have 7-fold improved long-term survival. Carcinogenesis 2015;36:76–81. 25380601 10.1093/carcin/bgu227 PMC4291047

[4] Pastorino S , Yoshikawa Y, Pass HI, Emi M, Nasu M, Pagano I, et al. A subset of mesotheliomas with improved survival occurring in carriers of BAP1 and other germline mutations. J Clin Oncol 2018;36:3485–94. 10.1200/JCO.2018.79.0352 PMC7162737 30376426

[5] Carbone M , Adusumilli PS, Alexander HR Jr, Baas P, Bardelli F, Bononi A, et al. Mesothelioma: scientific clues for prevention, diagnosis, and therapy. CA Cancer J Clin 2019;69:402–29. 31283845 10.3322/caac.21572 PMC8192079

